# XEN Glaucoma Implant for the Management of Operated Uncontrolled Glaucoma: Results and Complications during a Long-Term Follow-Up

**DOI:** 10.1155/2021/2321922

**Published:** 2021-07-09

**Authors:** Katarzyna Lewczuk, Joanna Konopińska, Joanna Jabłońska, Jacek Rudowicz, Patrycja Laszewicz, Zofia Mariak, Marek Rękas

**Affiliations:** ^1^Department of Ophthalmology, Military Institute of Medicine, Warsaw, Poland; ^2^Department of Ophthalmology, Medical University in Bialystok, M. Sklodowska-Curie 24A STR, 15-276 Bialystok, Poland

## Abstract

This study aimed to analyze the surgical and refractive outcomes of XEN glaucoma implant (Allergan, an Abbvie company, Irvine, CA, USA), a minimally invasive surgical device for the treatment of operated uncontrolled glaucoma. Eyes that received XEN Gel Stent placement from December 2014 to October 2019 were retrospectively investigated. Intraocular pressure (IOP) change, best-corrected visual acuity (BCVA), change in glaucoma medications, frequency of slit lamp revision procedures, and frequency of secondary glaucoma surgeries were the primary outcomes. Seventy-two eyes of 72 subjects were included in the study: 32 (44%) men and 40 (56%) women. The follow-up period ranged from 1 to 50 months (median, 26.13 months). The mean IOP before surgery was 24.82 ± 8.03 mmHg and decreased to 17.45 ± 5.84 mmHg at the end of the study (mean difference [MD] = −7.48, 95% confidence interval [CI]: −10.04, −4.93; *p* < 0.001). The mean decrease from baseline was 23%. BCVA before surgery was 0.38 ± 0.30, and that at the end of the follow-up period improved to 0.47 ± 0.37, MD = 0.09, 95% CI: 0.04, 0.13; *p* < 0.001. Additional procedures (fluorouracil injection and bleb needling) were performed in 11/72 patients (15%). Further glaucoma surgery was necessary for 23.9% of the patients. XEN Gel Stent implantation is both safe and reasonably effective for lowering IOP in operated uncontrolled glaucoma patients.

## 1. Introduction

Glaucoma remains the second leading cause of blindness worldwide [1], and the only known factor that can slow the progression of this disease is the reduction of intraocular pressure (IOP). Despite advanced pharmacological and surgical treatments, many cases of glaucoma progress to blindness [2]. Operated uncontrolled glaucoma (OUG) poses a challenge for glaucoma surgeons [3]. It is defined as an uncontrolled IOP with associated visual field deterioration, despite maximum tolerated antiglaucoma treatment and previously unsuccessful antiglaucoma procedures [4]. The term refractory glaucoma (RG) usually refers to the patients after at least 2 trabeculectomies with or without a cyclodestructive procedure. Moreover, OUG can occur in other patients with a high risk of fibrosis, such as those with a prolonged history of antiglaucoma medication use and ocular surface inflammation, as well as in patients of African descent. In clinical practice, refractory glaucoma cases usually present with neovascular glaucoma (NVG), congenital glaucoma or juvenile glaucoma (GJ), postinflammatory glaucoma (UG), traumatic glaucoma (TG), or glaucoma resulting from previous vitreoretinal procedures (oil-induced glaucoma).

In cases of operated uncontrolled glaucoma, use of glaucoma drainage devices (GDD) is often an interesting option. Their effectiveness is comparable to trabeculectomy; however, they present a relatively high risk of complications such as anterior chamber shallowing, choroidal detachment, filtering bleb leakage, blood in the anterior chamber, chronic corneal endothelial loss, hypotony, and macular edema [5,6]. In addition, GDD procedures are time-consuming and require great operating skill, especially in cases of conjunctival fibrosis wherein previous surgeries force the use of scleral quadrants other than the superior ones.

Therefore, it is imperative to search for new surgical methods that lower intraocular pressure safely and effectively. In 2016, the US Food and Drug Administration approved the new XEN® Gel Stent implant (XEN45 Gel Stent; Allergan plc, Dublin, Ireland) for the treatment of open-angle glaucoma (OAG), GJ, UG, and RG (CE certified in 2011). This implant belongs to the category of minimally invasive glaucoma surgery (MIGS) and its mode of action, like trabeculectomy and GDD, is to create a new route of outflow for the aqueous humor from the anterior chamber, bypassing the potential site of increased outflow resistance in the Schlemm canal (SC) and using the subconjunctival outflow route. Similar to trabeculectomy, it is a filtration-bleb-dependent procedure. This 6 mm long gelatin implant with internal cross-sections of 140, 63, and 45 *μ*m (in XEN140, XEN63, and XEN45, respectively) was designed based on the Hagen–Poiseuille law; depending on the length and cross-section, these devices have pressure differences along the implant [7]. It can be implanted with both the ab interno and ab externo approaches, with similar efficacy [8]). XEN® Gel Stent implant is constructed with a nonabsorbable, soft, cross-linked collagen tube, which is supposed to provide better biocompatibility, reduce inflammatory reactions to foreign bodies, and reduce fibrosis. It hydrates within 1-2 minutes (the external dimension increases, whereas the internal dimension remains unchanged), which enables the maintenance of the implant in the intended position without shifting and having to adapt to surrounding tissues. In contrast to trabeculectomy and GDD, the advantages of XEN® Gel Stent implant include microinvasive ab interno access, sparing of the sclera and conjunctiva, obviating the need for iridectomy and sutures, and a short procedure time.

The MIGS procedures are routinely performed in eyes with incipient or intermediate-stage glaucoma to reduce drug burden, or as an early intervention to lower the IOP without the use of a drug or laser [9]. However, XEN implantation is the only one among this category of procedures to use subconjunctival drainage of the aqueous humor—a pathway of aqueous humor outflow that has been the foundation of glaucoma surgery for over a century—in its mechanism of action. This advantage makes its use comparable with trabeculectomy, which is considered as the gold standard in glaucoma surgery, but is burdened with a high risk of complications often resulting in unpredictable outcomes and deterioration of vision [10]. Studies that compared the two treatments have demonstrated similar hypotensive efficacy with a better safety profile for XEN [11–13].

The efficacy of the XEN implant was demonstrated in intermediate and incipient glaucoma [14, 15]. However, only few studies have evaluated the efficacy of XEN implants in OUG [16–18], and the longest follow-up period in these studies was 12 months. Therefore, this study aimed to retrospectively evaluate the efficacy of XEN implantation in 72 patients with OUG during a long-term follow-up period.

## 2. Materials and Methods

This study was performed with approval from the Bioethics Committee of the Military Institute in Warsaw, in accordance with the ethical standards as laid down in the 1964 Declaration of Helsinki and its later amendments or comparable ethical standards. All study subjects gave written and informed consent for ophthalmological examination and the use of their clinical data for publication on the day of the first ophthalmological examination.

We retrospectively analyzed the data of patients who underwent XEN implantation for OUG, defined as IOP ≥21 mmHg, who had a history of previous unsuccessful antiglaucoma procedures, and who were subject to surgeries performed by the same experienced surgeon (MR) from December 2014 to March 2019. Inclusion criteria were defined as the follows: (1) age ≥ 18 years, (2) presence of OUG, defined as prior treatment with surgical or cyclodestructive procedures, and (3) failure to achieve target IOP with maximally tolerated topical IOP-lowering treatment or intolerance to drugs. Both primary and secondary open- and closed-angle glaucoma cases were included in this study. If a clinically significant cataract was also observed in a phakic patient, the patient qualified for a combined procedure with phacoemulsification and implantation of an artificial intraocular lens. Other inclusion criteria were presence of healthy mobile conjunctiva in at least one quadrant and best-corrected visual acuity (BCVA) better than light perception. Exclusion criteria were presence of clinically significant inflammation or infection within 30 days before surgery, history of corneal refractive surgery, corneal deposits or haze preventing intraoperative viewing of the anterior chamber, presence of an anterior chamber lens, advanced age-related macular degeneration (AMD), known or suspected allergy or sensitivity to porcine products or glutaraldehyde, pregnant or nursing women, and lack of consent to participate in the study. If patients were taking anticoagulants before surgery, they were discontinued under the supervision of a general practitioner, changed to low-molecular-weight heparin injections perioperatively, and then continued after surgery. If both eyes were eligible for surgery, the eye with the worse BCVA and visual field was operated first.

The number of previous surgical procedures was not an exclusion criterion.

During the procedure, depending on the availability of the surgical field, the surgeon used the ab interno or ab externo technique, following previously described guidelines [17, 19]. In cases where only the lower quadrants were accessible, the ab externo access was the technique of choice. All treatments were performed with 40 micrograms of mitomycin C, which was injected under the conjunctiva at least 6 mm from the corneal limbus in the projection of the future filtering bleb. The eyes were treated postoperatively with topical medication containing steroids (loteprednol; three times daily for four weeks, which was then tapered to BID for a week), an antibiotic (moxifloxacin; three times daily for two weeks), and nonsteroidal anti-inflammatory drugs (three times daily for 4 weeks) [20].

At the preoperative visit, the following information was obtained from the patient: age, sex, previous surgical procedures, BCVA (according to the Snellen chart), IOP (measured using Goldmann applanation tonometry), mean deviation of the visual field (assessed using the 24-2 algorithm of the Humphrey Visual Field Test), and the number of IOP-lowering medications used (counted as single substances or oral acetazolamide). For instance, 3 medications would be counted for an eye that was prescribed acetazolamide tablets and dorzolamide-timolol drops.

The number of antiglaucoma medications, IOP, and BCVA were analyzed before the surgery and at 1 day, 1 week, 1 month, 3 months, 6 months, 1 year, and 2 years postoperatively. From the day of the surgery, the patients had all antiglaucoma drugs discontinued, which were restarted according to the Advanced Glaucoma Intervention Study rule if the target IOP was not achieved after surgery [21]. The target pressure was achieved when no progression was observed. The final MD value at the end of the follow-up period was compared to that evaluated at the first visit.

Moreover, 5-fluorouracil (5-FU) injections, transconjunctival needling, and subsequent antiglaucoma surgeries were recorded as they occurred. Additional procedures were applied when the following criteria were met: for 5-FU injection (5 mg in 0.2 mL), progressive increase in the IOP greater than 16 mmHg on the consecutive visits and the development of subconjunctival fibrosis (manifested as engorged and tortuous blood vessels above filtering bleb); for needling, diagnosing of fibrosis (based on the abovementioned clinical signs), insufficient subconjunctival outflow, increase in the IOP, or the flattening of the bleb. Injections of 5-FU were administered for 5 consecutive days or until the fibrosis was abated and the IOP stabilized, provided that no antimetabolite-related adverse effects occurred [22, 23].

The number of complications, such as hypotony, choroidal detachment, corneal edema and keratopathy, improper positioning, leakage of the filtering bleb, implant displacement or occlusion, bleeding into the anterior chamber, malignant glaucoma, and intraocular inflammation, was noted. Hypotonia was defined as IOP ≤5 mmHg in two consecutive measurements at any stage of the follow-up period.

Complete surgical success was defined as a decrease of 20% in IOP, or IOP ≤15 mmHg without medication. Qualified success was defined as a decrease of 20% in IOP, or IOP ≤15 mmHg with up to 2 antiglaucoma medications.

Statistical analysis was performed using the R software, version 3.5.1. The study variables are presented using descriptive statistics. The normality of the distribution of quantitative variables was assessed using the Shapiro–Wilk test, data skewness, kurtosis indicators, and visual assessment of the histograms. The equality of variance was checked by Bartlett's test. Comparative analysis of the results between the beginning and the end of the study was performed with Student's *t*-test for dependent measurements. Mean difference (MD) with 95% confidence level (CI) was also calculated. Additionally, the cumulative incidence of complete success and cumulative incidence of satisfactory success were calculated using the Kaplan–Meier survival analysis. Missing values were omitted when analyzing individual variables. A significance level of *α* = 0.05 was used, and all tests were two-sided.

## 3. Results

### 3.1. Demographics and Glaucoma History

Seventy-two eyes of 72 subjects were included in the study (32 [44%] men and 40 [56%] women). The mean age of patients at the time of surgery was 59.51 ± 18.22 years (range, 23–88 years). The duration of the subjects' glaucoma ranged from 12 months to 56 years (mean, 14.38 ± 13.97 years). The mean follow-up period was 26.87 ± 15.33 months (median, 26.13 months; range, 6 to 50 months). Twenty-five patients (35%) had POAG, 12 (17%) had PXG, 8 (11%) had glaucoma associated with Axenfeld–Rieger syndrome or Cogan syndrome, 7 (10%) had silicone oil-induced glaucoma after vitrectomy, 6 (8%) had NG, 4 (5%) had UG, 3 (4%) had closed-angle glaucoma, 3 (4%) had secondary TG, 3 (4.5%) had secondary pigmentary glaucoma, and 1 (1.5%) had glaucoma after choroidal melanoma treatment with plaque brachytherapy. Ten patients underwent combined XEN implantation and phacoemulsification, while 62 received only XEN implantation.

The median number of prior surgeries performed was 1 (median 1; 2.25; range, 1–6). The prior treatments performed are shown in Table 1.

### 3.2. Intraocular Pressure

The mean IOP before surgery was 24.82 ± 8.03 mmHg and decreased to 17.45 ± 5.84 mmHg at the end of the study (MD, −7.48; 95% CI: −10.04, −4.93; *p* < 0.001). The mean decrease from baseline was 23% (Table 2).

### 3.3. Visual Acuity

**Figure 1:**
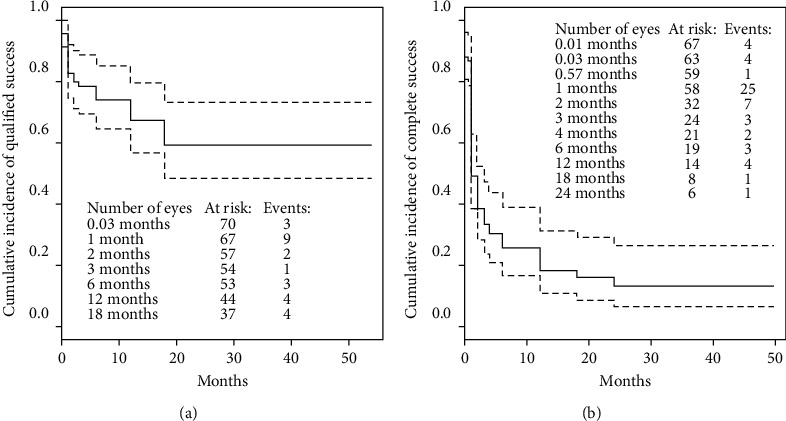
Kaplan–Meier cumulative incidence of qualified success and complete success. The dotted lines indicate 95% confidence interval.

BCVA before surgery was 0.38 ± 0.30, and that at the end of the follow-up period improved to 0.47 ± 0.37 (MD, 0.09; 95% CI: 0.04, 0.13; *p* < 0.001). The Kaplan–Meier cumulative incidence of qualified success was 59.4% after 24 months (95% CI: 48.2%, 73.1%), while the cumulative incidence of complete success after 2 years of observation was 13.4% (95% CI: 6.7%, 26.7%; Figure 1).

Complete surgical success was defined as a decrease of 20% in IOP, or IOP ≤15 mmHg without medication. Qualified success was defined as a decrease of 20% in IOP, or IOP ≤15 mmHg with up to 2 antiglaucoma medications.

At the end of the follow-up period, a decrease in BCVA, compared to the baseline value, was observed in 12/59 eyes (20%), while an increase was observed in 34/59 subjects (58%). The cases of decrease in BCVA were caused by posterior capsulae opacification (6 subjects—10%), development of AMD (3 subjects—5%), and progression of glaucomatous neuropathy (3 subjects—5%). On the other hand, the decrease in IOP level at the end of the study, relative to the baseline value, occurred in 49/64 subjects (77%); an increase in IOP was noted in 10/64 eyes (16%). A decrease in IOP equal to or greater than 20% occurred in 9/64 eyes (14.1%), representing 18% (9/49) of all eyes with a decrease in IOP. A final IOP level of less than 15 mmHg affected 31/64 (48%) eyes.

### 3.4. Visual Field

Visual Field of the mean defect (MD) before surgery was −19.0 ± 5.1. At the end of follow-up, stabilization was observed in 63.1% of the patients. Progression was observed in 36.9% of the patients.

### 3.5. Additional Interventions and Medications

Massage was recommended to 13/72 (18%) people, and needling was performed in 43/64 patients (67%); the median number of procedures per patient was 2 (interquartile range [IQR]: 1.5–3), with a range of 1 to 12. Subconjunctival 5-FU injection was required in 14/72 (19%) patients. The median number of patients who took medication before surgery was 4 (IQR: 3–5), with a range of 1 to 5, while the median number of patients who took medication after surgery was 2 (IQR: 1–4). The first drug was introduced after 49.5 days postoperatively (IQR: 20.5–137.5; Table 2).

**Figure 2:**
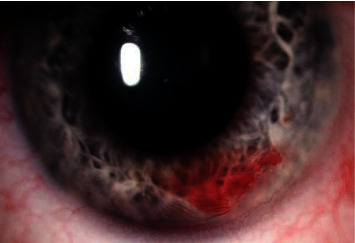
Complications of XEN implantation—bleeding into the anterior chamber.

**Figure 3:**
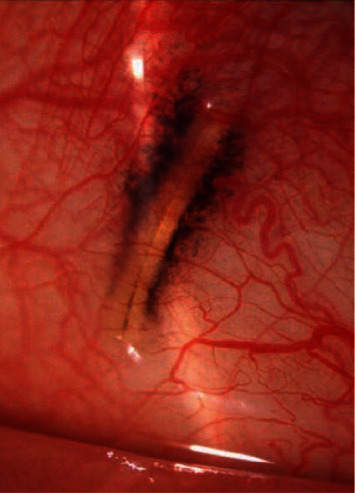
Complications of XEN implantation—protrusion under the conjunctiva.

**Figure 4:**
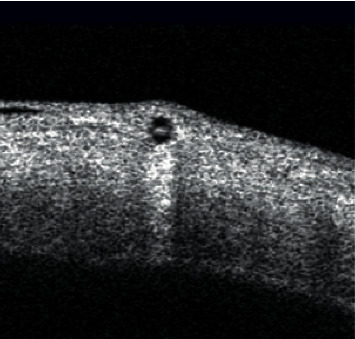
Complications of XEN implantation—an optical coherence tomography image showing protrusion under the conjunctiva.

**Figure 5:**
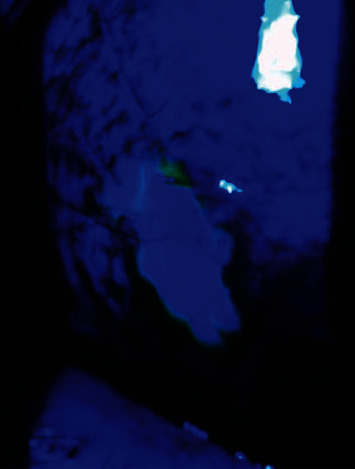
Complications of XEN implantation—bleb leakage.

**Figure 6:**
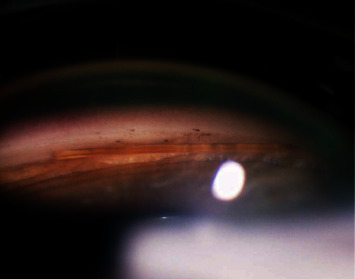
Complications of XEN implantation—protrusion of XEN into the anterior chamber.

Complications occurred in 23/69 patients (33%) (Figures 2–6, Tables 3 and 4), and reoperations were performed in 11/71 patients (24%).

The reoperated patients (11 eyes) had the following procedures: implantation of a second XEN in 6 and trabeculectomy in 3, and 2 eyes had bleb revision after XEN implantation. Two of these eyes underwent 2 subsequent procedures: the first one had a second XEN implanted 2 months after the procedure and trabeculectomy after another 10 months, and the second one underwent trabeculectomy 4 months after the procedure and implantation of a second XEN implant after another 7 months.

## 4. Discussion

The XEN® Gel stent implant is gaining more and more popularity among glaucoma surgeons as a natural alternative to trabeculectomy. To the best of our knowledge, only few clinical trials have evaluated its use in OUG cases. This retrospective analysis confirms that XEN implantation decreases both IOP and the number of antiglaucoma medications in patients with this type of glaucoma, with a relatively favourable safety profile.

Grover et al. [17] in their multicenter study on advanced glaucoma (14.3% of patients) and OUG (84.6% of patients) reported similar results in a group of 65 eyes over a period of 12 months. In his study, a total of 76.3% of the eyes in his study achieved surgical success in reducing IOP by ≥20% at 12-month follow-up when compared with the baseline value, with the same number or fewer medications than that before the surgery. In Grover et al.'s study, 84.6% of the patients also had previous surgery. A total of 38.5% of the patients in this study used no medications at the end of the follow-up period, and the mean number of medications dropped from 3.5 to 1.7 per patient. The most common complications in this study were the following: needling, a 2-line decrease in BCVA, transient hypotonia, and IOP spikes. No cases of intraocular inflammation were observed in the study by Grover et al. unlike our study, in which two cases of inflammation were reported. This is probably due to the surgical technique because all procedures were performed using the ab interno approach, similar to that used by Grover et al. The two cases of blebitis observed in our study occurred in the lower quadrants, which are at an increased risk of infection. However, both cases showed good response to treatment; after excision of the inflamed conjunctiva and antibiotic therapy, the blebitis resolved without any decrease in BCVA.

In the study, Tan et al. [24] presented an analysis of 39 cases with XEN® Gel stent implantation as the solo procedure with previous surgeries (30.8% cataract surgery, 7.7% trabeculectomy, and 5.13% iStent). Most of the patients in Tan et al.'s study had POAG (71%), while the remaining had PXG (5.1%), UG (10.3%), PDG (2.5%), NVG (2.5%), and steroid-induced glaucoma (2.5%). Initial IOP in this patient group was the same as that in our group (24.9 ± 7.8 mm Hg and 24.82 ± 8.03 mmHg, respectively), and the final IOP after 12 months of follow-up was lower than that in our group (14.5 ± 3.4 mmHg vs. 17.45 ± 5.84 mmHg, respectively); the number of medications decreased from 3 before the surgery to 0.7 after 12 months of follow-up (*p* < 0.005), when compared with that in our group, in which it was 4 before the surgery (IQR: 3–5) and 2 after the surgery (IQR: 1–4). The surgical success was higher at 62%, and the rate of complete and qualified success was 64% in Tan et al.'s study. All the previous studies have reported a more favourable hypotensive effect compared to our results; however, these studies had half the duration of the follow-up period as that in this study. In addition, given the stage of glaucoma in our study group, the target IOP was set at a relatively low level (15 mmHg), in contrast to other investigations, where an IOP of 18 mmHg was the criterion for surgical success.

This study has several limitations. First, it was a retrospective study, and assessments of patients, including IOP monitoring, were not masked and consequently subject to observer bias. A retrospective study uses existing data that have been recorded for reasons other than research and is generally considered inferior to prospective studies. Second, the study design lacked randomization and involved a probable selection bias. Third, inconsistent follow-up visits could have resulted in missed data points. Loss of statistical significance is a potential impact of loss to follow-up. Fourth, our study describes a group of cases that received an unusual treatment. However, based on our knowledge, this is the only study involving patients with a history of 1–5 antiglaucoma surgeries with a long-term follow-up period. It would be difficult to select a group for comparison with this material. Fifth, our study group was characterized by extremely diverse cases and different mechanisms of glaucoma in different patients. Finally, our study focused only on the Caucasian population. While this may be a study limitation on one hand, a homogeneous population is also advantageous as it allows the study of a specific ethnic group.

## 5. Conclusions

Our study has shown the mean decrease in IOP after XEN® Gel stent implantation of 23% in comparison to baseline. Postoperative IOP increase and the need for reoperation were the most common complications after surgery over a long-term follow-up period. To sum up, the applied surgery appears to show promising results in patients with OUG, owing to its minimal invasiveness. Pharmacoeconomic and quality-of-life studies in different groups of glaucoma patients are necessary to present this treatment method to patients as an alternative to trabeculectomy. As glaucoma remains the second most common cause of blindness, this study is clinically meaningful because it provides a better solution to the problem.

## Figures and Tables

**Table 1:** Previous surgery treatment.

Previous surgery	Number of eyes (%)
Trabeculectomy	73
Nonpenetrating deep sclerectomy	66
Cyclodestructive procedures	43
Canaloplasty	25
23G vitrectomy	21
Scleral buckling	12
ExPress seton implantation	10
Ahmed glaucoma valve implantation	8
Laser trabeculoplasty	65

**Table 2:** Visual acuity (decimal notification) and interocular pressure mean values, median values, standard deviations, and range before and after surgery.

	*n*	Mean (SD)	Median	Range	MD (95% CI)	*p*
*Visual acuity with the combined procedure (BCVA)*						
Preop	10	0.2 (0.1)	0.15	0.01 to 0.7	0.08 (0.02; 0.11)	0.001
Final	10	0.6 (0.22)	0.55	0.3 to 1.00		
Change	10	0.4 (0.44)	0.2	0.1 to 0.8		

*Visual acuity with the solo procedure (BCVA)*						
Preop	59	0.35 (0.31)	0.30	0.001 to 1.00	0.09 (0.04; 0.13)	<0.001
Final	49	0.41 (0.35)	0.40	0.00 to 1.00		
Change	49	0.08 (0.16)	0.01	−0.30 to 0.60		

*Intraocular pressure (IOP)*						
Preop	68	24.82 (8.03)	22.00	17.00 to 45.00	−7.48 (−10.04; −4.93)	<0.001
Final	64	17.45 (5.84)	17.00	7.00 to 38.00		
Change	64	−7.48 (10.24)	−5.00	−36.00 to 21.00		
Change (%)	64	−23.3% (32.5%)	−24.2%	−81.6% to 123.5%		

SD: standard deviation; preop: preoperatively; MD: mean difference calculated as final value–preop value with 95% confidence interval; paired *t*-test; combined procedure: XEN implantation with phacoemulsification; solo procedure: XEN implantation.

**Table 3:** Clinical characteristics of the study group.

Characteristic	*N*	%	Median (Q1; Q3)	Range
*BCVA change in the combined procedure group, n (%)*				
Decline	1/10	10		
No change	1/10	10		
Increase	8/10	90		

*BCVA change in the solo procedure group, n (%)*				
Decline	12/59	20.3		
No change	13/59	22.0		
Increase	34/59	57.6		

*IOP change, n (%)*				
Decline	49/64	76.6		
No change	5/64	7.8		
Increase	10/64	15.6		
Decline ≥ 20%	9/64	14.1		
Decline < 20%	40/64	62.5		
IOP final level ≤ 15 mmHg	31/64	48.4		
Massage recommendation, *n* (%)	13/72	18.0		
Needling, *n* (%)	43/64	67.2		
Number of needling procedures	43		2.00 (1.50; 3.00)	1–12
Number of drugs before surgery	67		4.00 (3.00; 5.00)	1–5
Time to inclusion of first drug, days	54		49.50 (20.50; 137.50)	0–764
Final number of drugs	67		2.00 (1.00; 4.00)	0–4
Complications, *n* (%)	23/69	33.3		
Reoperations, *n* (%)	17/71	23.9		

Combined procedure: XEN implantation with phacoemulsification; solo procedure: XEN implantation.

**Table 4:** Postoperative complications.

Postoperative complications	*n*	%
Dellen	1	1.4
Malignant glaucoma	1	1.4
Bleeding under the choroid	1	1.4
Migration of the implant into the anterior chamber	1	1.4
Filtering bleb leakage	1	1.4
Implant occlusion	1	1.4
Bleeding into the anterior chamber	2	2.8
Incorrect placement of the implant tip under the conjunctiva	2	2.8
Corneal edema and keratopathy	2	2.8
Corneal epithelial defects and erosion	2	2.8
Blebitis	2	2.8
Recurrence of uveitis	3	2.8
Implant extrusion	3	4.1
Serous choroidal detachment	3	4.1
Hypotonia	4	5.5
Revision/another antiglaucoma surgery	11	15.2
Increase in IOP > 21 mmHg	35	58

## Data Availability

Readers can access the data supporting the conclusions of the study upon an e-mail request on corresponding author. The names and personal data of the participants cannot be released due to ethical aspects.
